# Supplementary material for: Porcine Reproductive and Respiratory Syndrome Virus Engineered by Serine Substitution on the 44th Amino Acid of GP5 Resulted in a Potential Vaccine Candidate with the Ability to Produce High Levels of Neutralizing Antibody
Source: Vet Sci. 2023 Mar 3;10(3):191. doi: 10.3390/vetsci10030191 (PMC10055445; doi:10.3390/vetsci10030191)
Supplement: Supplementary file 1 [file vetsci-10-00191-s001.zip › Table S1.pdf]

Table S1. Level of viremia after inoculation with vCSL1-GP5-N44S. RNA was extracted from the serum obtained 0, 1, 3, 7, 14, 21, 28, 35, and 42 d post-inoculation; the virus titer in the serum was measured using the RT-qPCR technique.

Group	Level of Viremia (logTCID$_{50}$/mL Equivalent) at Post-Inoculation Day								
	**0**	**1**	**3**	**7**	**14**	**21**	**28**	**35**	**42**
Group 1 (vCSL1-GP5-N44S)	0.00	0.00	0.00	0.00	0.00	0.00	0.00	0.00	0.00
Group 2 (negative control)	0.00	0.00	0.00	0.00	0.00	0.00	0.00	0.00	0.00
