# Supplementary material for: Porcine Reproductive and Respiratory Syndrome Virus Engineered by Serine Substitution on the 44th Amino Acid of GP5 Resulted in a Potential Vaccine Candidate with the Ability to Produce High Levels of Neutralizing Antibody
Source: Vet Sci. 2023 Mar 3;10(3):191. doi: 10.3390/vetsci10030191 (PMC10055445; doi:10.3390/vetsci10030191)
Supplement: Supplementary file 1 [file vetsci-10-00191-s001.zip › Table S2.pdf]

Table S2. Clinical score after infection with the tentative hypoglycosylated mutant virus. Every 0, 1, 3, 7, 14, 21, 28, 35, and 42 d post-inoculation, the degree of clinical signs was measured according to the criteria of the previous study.

Group	Clinical Score on the Post-Inoculation Day (Respiratory Score/Activity and Depression Score)								
	**0**	**1**	**3**	**7**	**14**	**21**	**28**	**35**	**42**
Group 1 (vCSL1-GP5-N44S)	0/0	0/0	0/0	0/0	0/0	0/0	0/0	0/0	0/0
Group 2 (negative control)	0/0	0/0	0/0	0/0	0/0	0/0	0/0	0/0	0/0
