# Supplementary figures and images for: Porcine Reproductive and Respiratory Syndrome Virus Engineered by Serine Substitution on the 44th Amino Acid of GP5 Resulted in a Potential Vaccine Candidate with the Ability to Produce High Levels of Neutralizing Antibody
Source: Vet Sci. 2023 Mar 3;10(3):191. doi: 10.3390/vetsci10030191 (PMC10055445; doi:10.3390/vetsci10030191)

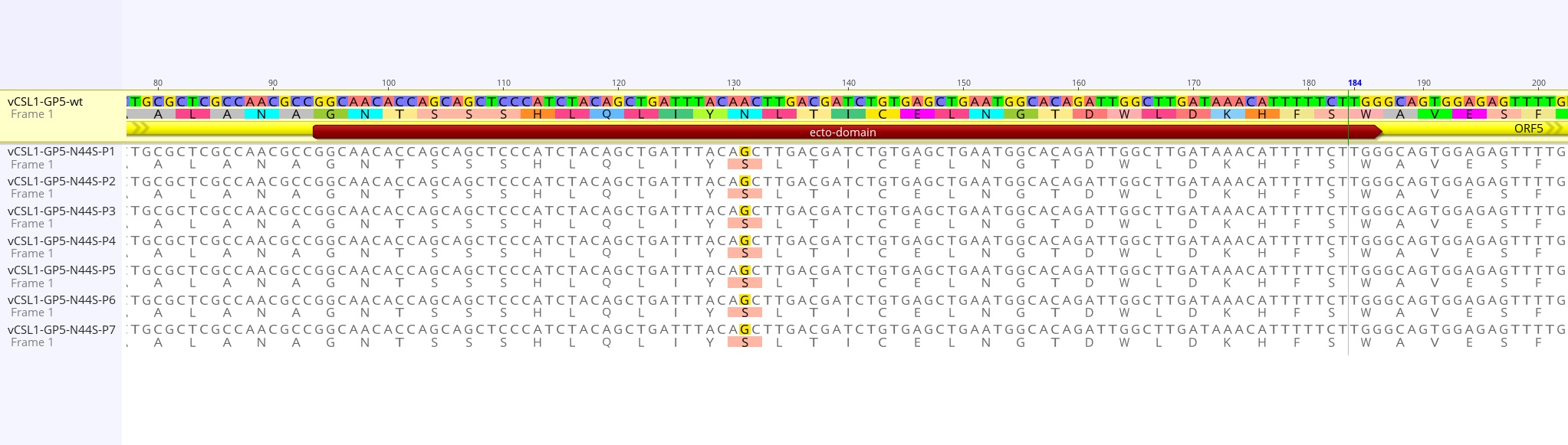

Supplement: Supplementary file 1 [file vetsci-10-00191-s001.zip › Figure S1.jpg]
